# The Economics Spectrum Drives Root Trait Strategies in Mediterranean Vegetation

**DOI:** 10.3389/fpls.2021.773118

**Published:** 2021-11-23

**Authors:** Enrique G. de la Riva, José Ignacio Querejeta, Rafael Villar, Ignacio M. Pérez-Ramos, Teodoro Marañón, Javier Galán Díaz, Sergio de Tomás Marín, Iván Prieto

**Affiliations:** ^1^Department of Ecology, Brandenburg University of Technology, Cottbus, Germany; ^2^Departamento de Conservación de Suelos y Agua, Centro de Edafología y Biología Aplicada del Segura – Consejo Superior de Investigaciones Científicas (CEBAS-CSIC), Murcia, Spain; ^3^Área de Ecología, Departamento de Botánica, Ecología y Fisiología Vegetal, Facultad de Ciencias, Córdoba, Spain; ^4^Institute of Natural Resources and Agrobiology of Seville (IRNAS), CSIC, Seville, Spain; ^5^Doñana Biological Station (EBD), CSIC, Seville, Spain; ^6^Departamento de Ecología, Facultad de Biología y Ciencias Ambientales, Universidad de León, León, Spain

**Keywords:** ecophysiology, fine root strategies, non-mass normalized traits, root anatomy, root economic spectrum, specific root area, specific root length

## Abstract

Extensive research efforts are devoted to understand fine root trait variation and to confirm the existence of a belowground root economics spectrum (RES) from acquisitive to conservative root strategies that is analogous to the leaf economics spectrum (LES). The economics spectrum implies a trade-off between maximizing resource acquisition and productivity or maximizing resource conservation and longevity; however, this theoretical framework still remains controversial for roots. We compiled a database of 320 Mediterranean woody and herbaceous species to critically assess if the classic economics spectrum theory can be broadly extended to roots. Fine roots displayed a wide diversity of forms and properties in Mediterranean vegetation, resulting in a multidimensional trait space. The main trend of variation in this multidimensional root space is analogous to the main axis of LES, while the second trend of variation is partially determined by an anatomical trade-off between tissue density and diameter. Specific root area (SRA) is the main trait explaining species distribution along the RES, regardless of the selected traits. We advocate for the need to unify and standardize the criteria and approaches used within the economics framework between leaves and roots, for the sake of theoretical consistency.

## Introduction

Fine roots perform multiple essential functions, including acquisition and storage of soil resources (McCormack and Iversen, 2019). The variation in functional traits of fine roots has become an uprising research topic in the last years aimed at enhancing our understanding of the large diversity of plant belowground uptake strategies (Freschet et al., 2021). Functional trait variation often represents different plant strategies or adaptations to a wide range of environmental conditions (Wright et al., 2004; Díaz et al., 2016), which usually respond to a trade-off between growth and survival (Reich, 2014). This implies that plants invest in their attributes accordingly to an overarching trade-off between maximizing resource acquisition and productivity or maximizing resource conservation and longevity, the so-called “fast-slow” economics spectrum (Wright et al., 2004; Reich, 2014). This theoretical framework has been successfully linked to plant performance at the leaf level (Wright et al., 2004) but still remains controversial for roots (Freschet et al., 2021).

In the last years, large databases have been built to compile and analyze root trait variation globally (Freschet et al., 2017; Iversen et al., 2017). A search within the largest root database to date (GRoot; Guerrero-Ramírez et al., 2020) showed that the most commonly measured morphological and chemical root traits are specific root length (SRL; measured in 31.7% of all the species in the database), root nitrogen concentration (RN; 27.6%), root diameter (Rdi; 26.0%), and root tissue density (RD; 23.6%), for which their roles in plant functioning and resource economics have been relatively well described (Freschet et al., 2021). By analogy with leaves, plant species growing in more productive environments would have thinner roots with lower tissue density and higher SRL, which enhance a faster return of investments by favoring a higher metabolic activity and respiration at the expense of lower longevity. By contrast, in resource-poor environments the opposite trend of root traits is frequently observed (Roumet et al., 2016; de la Riva et al., 2018). Despite this general trend of root trait covariation, contrasting results have been reported in the literature. Several studies support the existence of a main trend of root trait covariation in line with the root economics spectrum (RES) theory (Prieto et al., 2015; Roumet et al., 2016; de la Riva et al., 2018), whereas other approaches advocate for a multidimensionality of root uptake strategies (Valverde-Barrantes et al., 2015; Weemstra et al., 2016). Recently, evidence supporting a bivariate plane of root specialization called “root economics space” has emerged (McCormack and Iversen, 2019; Bergmann et al., 2020). Within this bidimensional space, the second axis of variation represents the classical trade-off between fast and slow resource uptake strategies (RES, *sensu* Bergmann et al., 2020), while the first axis is defined as a “collaboration” gradient that ranges from species with high SRL and short lifespan that do not depend on mycorrhizal fungi for water and nutrient uptake (“do-it yourself” strategy) to roots with a higher investment in mass per unit of root length (low SRL) that outsource resource uptake *via* symbiotic associations with mycorrhizal fungi as a means to enhance nutrient acquisition. This “outsourcing” strategy has morphological and functional consequences for the roots, as plants with larger Rdi depend more heavily on mycorrhizal fungi for an efficient resource acquisition. However, there is not unanimous agreement about the mechanisms underlying the costs of these root modifications. Thus, a clear explanation for how and why some key fine-root traits sometimes co-vary in a coordinated manner but at other times appear to vary independently is still missing (McCormack and Iversen, 2019).

The lack of consistency in root trait covariation brings up another key aspect that warrants further research, the often reported mismatch between economics spectrum of leaf and root traits. In leaves, the leaf economics spectrum (LES) was defined as the main trend of variation of mass-normalized morphological and physiological traits (e.g., leaf mass per area, photosynthetic capacity), leading to a trade-off between resource acquisition and conservation that is widely supported at a global scale (Wright et al., 2004; Díaz et al., 2016). This resource-use paradigm has helped to explain leaf trait covariation across species and has been successfully linked to plant strategies both globally and at regional and local scales (Wright et al., 2004; Reich, 2014; Prieto et al., 2018). In parallel, extensive efforts are being carried out to better understand the role of root trait variation and to confirm the existence of a belowground RES, analogous to the LES (Prieto et al., 2015; Roumet et al., 2016; de la Riva et al., 2021). However, the LES and the RES are not always coordinated with each other, and one of the main reasons for these discrepancies could be a potential mismatch between the criteria and traits used to define each of them. Plants show strong multidimensional variation in their “non-mass normalized” attributes whether for leaves or roots [i.e., leaf thickness (Lthick) or root diameter]. However, the widely accepted perspective in the LES approach is that, despite the important roles of leaf tissue density (LD), thickness, area and hydraulic (i.e., stomatal density) or physiological traits (leaf δ^13^C and δ^18^O) to understand the structure and functioning of leaves (Poorter et al., 2009; Wright et al., 2017; Prieto et al., 2018; Bertolino et al., 2019), neither of these traits are normally more integrative than the specific leaf area (SLA; leaf area per unit of dry mass) or its inverse leaf mass area (LMA; Hodgson et al., 2011; Onoda et al., 2011), which have been considered central attributes of plant strategies. Interspecific variation in SLA is determined by modifications in its underlying components, Lthick and density (Villar et al., 2013; Gratani et al., 2018). However, despite the trade-off between these two components modulating SLA variation, the LES is considered the main trend of functional variation in leaves (Poorter et al., 2009; de la Riva et al., 2016). In contrast to leaves, both mass- and non-mass-based root traits have been frequently and inconsistently analyzed as components of the functional resource uptake strategies, being SRL and its underlying components (RD and diameter) interpreted as a dimension of root variation that is different from the classic economics spectrum, based on mass-normalized traits (i.e., Kramer-Walter et al., 2016; Bergmann et al., 2020). In addition, the historical lack of robust root trait data (Iversen et al., 2017) and the absence of studies considering morphological and physiological traits simultaneously across a large number of species (as can be observed in the GRoot database; Guerrero-Ramírez et al., 2020), has made it very difficult to assert the generality of the RES at the same level as the LES. Therefore, a more precise and rigorous definition of the RES would help to develop a general theory that explains root trait coordination and trade-offs in a wider array of different contexts, using the fewest but most critical attributes of the belowground plant fraction (Reich, 2014).

Based on this background, the present study aimed to explore root trait variation in a large pool of Mediterranean species and assess the main trends of belowground trait diversity under Mediterranean conditions. In a recent study, we have shown evidence that the currency of root economics is the amount of photosynthates required to construct fine roots (root construction costs) that explore the soil for resource acquisition. We observed a main trend of root trait variation, mainly determined by the covariation between specific root area (SRA), root dry matter content (RDMC), and root density (RD) that showed a clear relationship with root construction costs (de la Riva et al., 2021). Here, we advocate for a more rigorous definition of the main attributes of the economics spectrum, at the root level, that more closely matches the approach used for leaves. This approach will help to define the main planes of root trait variation to better understand which are the most integrative plant strategies encompassing above- and belowground traits. This approach will also help to build a starting point to integrate the complexity of the belowground component in a main trend of root trait variation that defines different resource acquisition-conservation strategies. To do this, we focus on three central hypotheses relevant to the economics spectrum theory in order to evaluate whether it can be broadly extended to roots.

1. The RES is the main trend of root trait covariation in Mediterranean species.
2. Specific root area is a more informative trait than SRL from a plant economics perspective (PES).
3. An anatomical trade-off between tissue density and thickness/diameter exists for both leaves and roots.

To explore these questions, we have compiled a dataset of functional traits with 320 species from Mediterranean – climate areas of the Iberian Peninsula (119 woody and 201 herbaceous) measured using the same protocol. The review and analysis of this dataset will hopefully help to clarify the abovementioned questions and can be a useful complement to the many earlier works it builds upon.

## Materials and Methods

### Database

The analyses presented in this manuscript are based on 532 observations from 320 species (119 woody and 201 herbaceous species) with a large phylogenetic diversity (32 different taxonomic orders). The Mediterranean biome is one of the most diverse in the world, with a wide range of environments and plant adaptations; thus, our dataset includes species from arid to sub-humid Mediterranean areas (Appendix S1). More importantly, these 320 species encompass *ca.* 95% of the range of mean variation in LMA encountered globally (Poorter et al., 2009).

To build this database, we selected studies or unpublished data carried out during the last decade by the authors (see Appendix S1; de la Riva et al., 2016, 2021 for more details) that contained measurements of eight root (five morphological and three chemical) and six leaf (four morphological and two chemical) functional traits linked to the root- and leaf economics spectrum, respectively. One strength of the database is that leaves and roots were collected on individual trees following the same sampling protocol. For fine roots (<2mm), we selected SRL (m g^−1^), SRA (m^2^ kg^−1^), RDMC (g g^−1^), RD (g cm^−3^), Rdi (mm), and the concentration of carbon (RC), nitrogen (RN), minerals, and organic N. For leaves, we selected SLA (m^2^ kg^−1^), leaf dry matter content (LDMC; mg g^−1^), Lthick (mm), leaf tissue density (LD, calculated as the ratio of LMA and thickness; g ml^−1^), and the concentration of leaf nitrogen (LN). In total, we analyzed information of 320 species for root morphological traits (SRL, SRA, RDMC, RD, and Rdi) and for 314 species for leaf morphological traits (SLA, LDMC, Lthick, and LD). Information for leaf and root chemical traits (N and C) was obtained from 64 woody species (from Marañón et al., 2020; de la Riva et al., 2021; 80 observations in total) and minerals and organic N concentration was obtained from 60 species (from de la Riva et al., 2021; 73 observations).

### Data Analyses

All data processing and analyses were done using R 3.6.1. To obtain an overview of the dimensional variation of root and leaf traits (hypothesis 1), we conducted two separate principal component analyses (PCA), one for roots and one for leaves. We used a subset of analogous morphological traits linked to economics spectrum at root (SRL, SRA, RDMC, Rdi, and RD; de la Riva et al., 2021) and leaf level (SLA, LDMC, LD, and Lthick), respectively for the leaf and root PCAs. For these PCAs, and in order to use all the measurements in the database (532), when trait measurements for a given individual were not complete (e.g., there were missing values for one trait, which happened in 15 individuals in total and only for leaf traits), we calculated the mean value for that given trait using the function: apply {dataset, 2, function (x) ifelse [is.na(x), mean(x, na.rm.=TRUE), x]}. Additionally, we carried out two separate PCAs for woody and herbaceous species, respectively, which allowed us to assess the influence of the growth forms in the observed patterns.

To control for potential phylogenetic effects on trait covariation, we conducted the same PCAs (leaves and roots and herbaceous and woody species) using phylogenetically independent contrasts and a mean trait value per species for 318 Mediterranean plant species (two ferns species *Equisetum ramosissimum* and *Pteridium aquilinum* were excluded from the analysis because their phylogenetic information was not available in the ALLMB tree used to build the phylogenetic tree for our set of species). We used the “phyl.pca” function implemented in the library RPANDA (Morlon et al., 2016) to conduct the phylogenetically informed PCAs. Information to construct the phylogenetic tree of the studied species was obtained from the ALLMB tree (Smith and Brown, 2018; available in https://github.com/FePhyFoFum/big_seed_plant_trees). The phylogenetic distance of the species (52 in total) that were not found in the ALLMB database were supplanted by the distance of the closest species of the same genus found in the mega-phylogeny tree (see de la Riva et al., 2019 for more details).

To test if the SRA is better proxy than SRL (hypothesis 2), the degree of coordination among SRA and SRL with chemical root traits was determined with Pearson correlations coefficients.

To observe the covariation between SRA with RD and RDi and SLA with LD and Lthick (hypothesis 3), we first run two separate PCAs for each plant organ (leaves and roots) with these specific traits and the total pool of species (532 observations). Second, we selected species for which root nitrogen was available (64 woody species, 80 observations). We then run three different PCAs in order to compare the patterns obtained within our dataset with those related with the root economic space as defined by Bergmann et al. (2020): (I) a PCA with the same traits proposed by Bergmann et al. (2020); (II) the same PCA, where we replaced SRL by SRA; and (III) the PCA proposed by Bergmann et al. (2020) using analogous leaf and root traits (SRA, RD, and Rdi and SLA, LD, and Lthick).

## Results

### The Root Economics Spectrum Is the Main Trend of Variation in Mediterranean Plants

**Figure 1:**
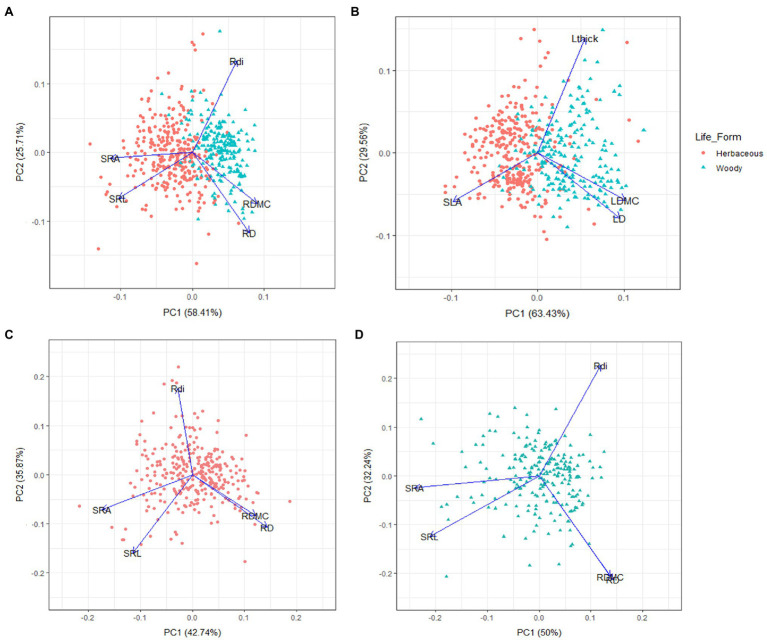
Plot of the first and second axes of the principal component analysis (PCA) for the total dataset (532 observations from 320 species) performed with: root morphological traits **(A)**; the analogous leaf morphological traits **(B)**; root morphological traits only for woody **(C)**; and herbaceous plants **(D)**. SRL, specific root length; SRA, specific root area; RDMC, root dry matter content; RD, root tissue density; SLA, specific leaf area; LDMC, leaf dry matter content; LD, leaf tissue density; Rdi, root diameter; Lthick, leaf thickness.

The results of the general PCA for the 532 observations from 320 species are shown in Figure 1. The first PCA axis (58.4% of total trait variation) represents a gradient from SRA and SRL to RDMC and RD, confirming certain coordination among these four root morphological traits (Figure 1A). The second PCA axis (25.7% of total trait variation) was mostly determined by the variation in Rdi (Figure 1A). Similarly, the PCA of leaf showed the same dimensions of variation as root (Figure 1B). That is, the first principal component (PC; 63.4%) was explained by one extreme with high values of SLA, and at the opposite extreme with high values of LDMC and density (LD), while the second PC axis (29.5%) was mainly represented by Lthick variation. When including phylogenetic independent contrast (PIC) in the PCA, the patterns for root and leaf traits were consistent (Appendix S2), further confirming that species evolutionary history did not influence the observed patterns. Despite the strong segregation between growth forms along the PCA at root level (herbaceous vs. woody species; one-way ANOVA with the PCA scores of the first component, value of *p*<0.001), the root PCAs for each growth form showed similar dimensions of variation as in the total dataset (Figures 1C,D), with high scores for their respective first PCs (42.7 and 50% of overall variation explained for woody and herbaceous plants, respectively).

### SRA Is a More Informative Trait Than SRL From a Plant Economics Perspective

The Pearson’s correlation analyses among morphological and chemical root traits from the subset of woody plants showed that both SRL and SRA were significantly correlated with chemical root traits (value of *p*<0.05; Table 1), such as carbon (C), nitrogen (N), organic Nitrogen (OrgN), and mineral concentration (MinCon). However, the SRA showed higher values of Pearson correlation coefficients than SRL in all cases (Table 1).

**Table 1:** Pearson correlation coefficients for relationships of specific root length (SRL) and specific root area (SRA) with chemical root traits (RN, root nitrogen concentration; RC, root carbon concentration; OrgN, organic nitrogen concentration; and MinCon, mineral concentrations).

	SRL		SRA	
	*r*	*p*	*r*	*p*
RN^1^	0.22	**0.03**	0.39	**<0.001**
RC^1^	0.28	**0.009**	0.54	**<0.001**
OrgN^2^	0.28	**0.01**	0.48	**<0.001**
MinCon^2^	0.41	**<0.001**	0.64	**<0.001**

Data from Marañón et al. (2020) and de la Riva et al. (2021).

1 80 observations from 64 woody species.

2 73 observations from 60 woody species.

### There Is an Anatomical Trade-Off Between Tissue Density and Diameter/Thickness

**Figure 2:**
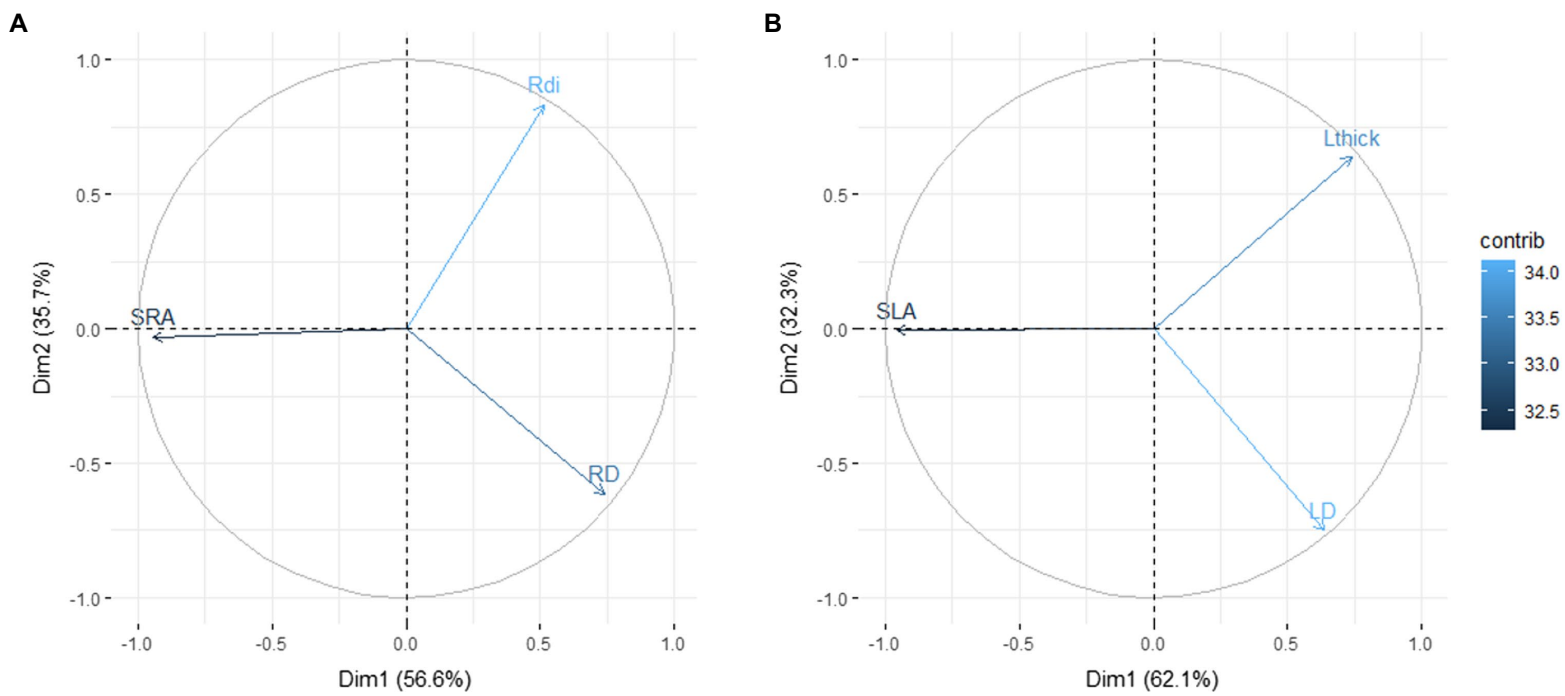
Plot of the first and second axes of the principal component analysis (PCA) demonstrating the trade-off between the specific area per unit mass (SRA/SLA) with tissue density (RD/LD) and diameter/thickness (Rdi/Lthick) at root **(A)** and leaf levels **(B)** for 561 observations from 310 Mediterranean plant species. Abbreviations are as in Figure 1.

Regarding the links between SRA and SLA with tissue density and root diameter or leaf thickness, the PCAs with the total dataset showed exactly the same trait distribution in both cases (Figure 2). Thus, the first axes of both PCAs were mainly determined by the SRA and SLA variation, respectively, while the second axes were defined by the trade-off between tissue density with diameter (in root) and thickness (in leaf).

**Figure 3:**
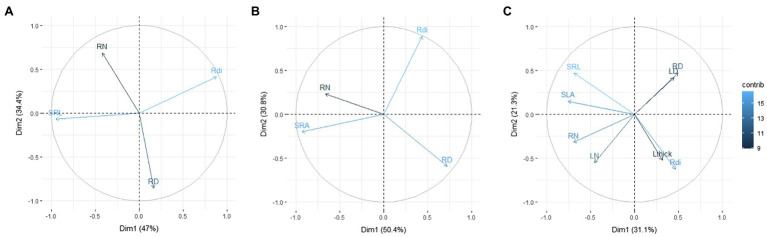
Plot of the first and second axes of the PCA from 80 observations of 64 Mediterranean woody species **(A)** with morphological traits used in Bergmann et al. (2020); **(B)** same PCA as in A but with SRA instead of SRL; and **(C)** PCA with analogous root and leaf morphological traits. Trait abbreviations are as in Figure 1 and Table 1. Data from de la Riva et al. (2021).

By testing the trait distribution proposed by Bergmann’s model with the subset of woody species, in which we have nitrogen concentration at leaf and root level (80 observations of 64 species), we confirmed the bivariate relationships underlying a trait space with two main dimensions (Figure 3A). The first PCA axis represents the negative correlation between SRL and Rdi, defined by the authors as “collaboration gradient,” while the second axis was defined by the variation between RD and root N, defined by the authors as “conservation gradient.” However, when SRL was replaced by SRA, the main PCA axis was representative of the resource uptake strategy, with higher values of SRA and RN at one end and RD at the other; whereas the second axis reflected the trade-off between density and diameter. Similarly, when the analogous root and leaf traits are included in Bergman’s model, the main PCA axis was mainly defined by the resource uptake strategy (species with higher SLA, SRL, RN, and LN in one end and species with higher LD, RD, Lthick, and Rdi at the other end), highlighting the strong coordination among analogous traits, while the second PCA axis did not show a clear pattern (Figure 3C).

## Discussion

### The Root Economics Spectrum Is the Main Trend of Variation in Mediterranean Plants

Wright et al. (2004) defined as a criterion for the variation along the first PCA axis of the LES that traits should follow (…*the same directionality of trait loadings and similarly high percentage of variance explained by the principal axis*). Following this criterion, the first PCA axis in our dataset represents a main trend of trait covariation with most of the total variance explained by this axis, showing a clear trade-off in the morphological structure of roots in Mediterranean species strongly dominated by mass-normalized traits that we can define as the RES. At the positive end of this axis, we find mainly woody species with high RDMC and low SRA and length (SRL), a syndrome commonly associated with a resource-conservation strategy. At the other end of the specialization gradient, we find mostly herbaceous species with opposite traits, a syndrome frequently associated to resource-acquisitive strategies (Roumet et al., 2016; de la Riva et al., 2018). We obtained similar results when taking into account phylogenetic effects in the PCA. Despite large differences in root trait values between herbaceous and woody species, we found remarkably similar patterns within these life forms separately. This evidence supports that the RES is a general syndrome that holds across and within life forms, at least under Mediterranean conditions.

Defining the RES as a dominant axis of root trait covariation has been challenging (i.e., Kramer-Walter et al., 2016; Weemstra et al., 2016; Bergmann et al., 2020) and the underlying reasons for the discrepancies found across studies, species or environmental gradients are still not clear. For example, Laughlin et al. (2017) evidenced that ontogenetic and allometric constraints related to divergent responses of traits within species growing across a range of environments may affect the strength of trait coordination across species. Other authors (Shen et al., 2019 and references therein) have suggested that some traits could be constrained by environmental factors, not necessarily related to resource uptake and the economics spectrum (Kramer-Walter et al., 2016; Weemstra et al., 2016) resulting in weak or inconsistent relationships among traits across environments. Another key factor underlying these discrepancies could be that most of these studies on root trait variation have frequently mixed up “non-mass-” and “mass-normalized” traits to develop the root economics spectrum paradigm (i.e., Weemstra et al., 2016), an aspect that we discuss further later.

### SRA Is a More Informative Trait Than SRL From a Plant Economics Perspective

Specific root length is probably one of the most commonly measured traits to characterize the economics spectrum at the belowground level (Ostonen et al., 2007). SRL is the ratio between root length and root dry mass (Ryser, 2006); root length is assumed to be proportional to resource acquisition (foraging or exploitation capacity), while root dry mass should be proportional to construction and maintenance costs (Eissenstat and Yanai, 1997). Hence, thinner and longer roots (higher SRL) are considered as the equivalent of thinner leaves (high SLA), which are less expensive to construct (Villar and Merino, 2001; Villar et al., 2006). In fine roots, SRL is often positively related to root respiration rates, the amount and rate of water and nutrient uptake, and plant growth rates (Eissenstat and Yanai, 1997; Reich et al., 1998; Tjoelker et al., 2005; Roumet et al., 2016). Therefore, and analogously to SLA in leaves, SRL has been commonly accepted as a key trait to screen and characterize plants according to their ecological strategy and productivity (Freschet et al., 2021).

However, the relationship between SRL and root functioning is not always clear; a fact that often hinders its interpretation and could raise questions for its use as a key explanatory trait of the RES. For example, Eissenstat et al. (2000) did not find any relationship between SRL and root physiology or root lifespan in grasses; Bergmann et al. (2020) found no relationship between SRL and RN or root life span globally, which were instead strongly related to root tissue density. In the late 90s, Ryser (1996, 1998) already argued that from a cost–benefit perspective this mismatch between SRL and root chemical traits is difficult to explain. Within our dataset, we observed contrasting relationships among anatomical variables involving root length (SRL) or root area (SRA), which could shed some light and explain this apparent contradiction (see details below). One possible explanation is that variations in SRL are tightly coupled with variations in root diameter (McCormack and Iversen, 2019; Bergmann et al., 2020) while variations in root surface area (SRA) are mainly determined by variations in root tissue density (de la Riva et al., 2018). It is frequently assumed that higher SRL enhances soil volume exploration per unit dry mass with a small associated metabolic cost (Ryser, 2006) and that the volume of soil within the influence of a given root is a more important factor than the root surface area; therefore, SRL has been considered a better proxy for resource uptake than SRA (Freschet et al., 2021). However, an increase of the total absorptive surface area per unit mass for a given length in roots should increase the potential to encounter relatively immobile soil resources (McCormack and Iversen, 2019), which may increase the belowground resource uptake capacity. This would be analogous to leaves with high SLA that increase the interception of photosynthetically active radiation to enhance C assimilation. We acknowledge that our studies are observational and, therefore, we cannot provide a mechanistic explanation for the advantage of SRA over SRL as a better proxy of the RES. However, based on 532 observations from 320 species with complete root trait information (SRA and SRL) and the same sampling protocol, we are able to show here that SRA is the main trait explaining the species distribution along the RES, regardless of the selected traits, plant functional groups or phylogenetic effects. In addition, for Mediterranean vegetation SRA is more tightly correlated than SRL with the dry matter content and chemical composition of both roots and leaves (de la Riva et al., 2018, 2021; Marañón et al., 2020); two features strongly related to metabolic rates and life span (Prieto et al., 2015; Roumet et al., 2016). In a recent study, we were able to show that root construction costs (the currency for root economics) were related to SRA but not to SRL, indicating that area-based rather than length-based traits may provide a better proxy of the investment per unit mass belowground (de la Riva et al., 2021). Unfortunately, SRA is a trait rarely reported in the literature: for instance, SRA was reported only in 7% of the species in the GRoot database (Guerrero-Ramírez et al., 2020), so we are unable to extend this hypothesis to other ecosystems. Therefore, further studies should be conducted in different ecosystem types to examine the role of SRA as a key trait in belowground resource economics (RES). We advocate that SRA is more informative than SRL as a general measure of root resource use efficiency in Mediterranean species, which is consistent with the observed role of its aboveground equivalent SLA as a key trait in leaf economics (Wright et al., 2004; Reich, 2014). However, our proposal by no means neglects the important roles of other root attributes, as SRL, to better understand belowground structure and functioning.

### There Is an Anatomical Trade-Off Between Tissue Density and Diameter/Thickness

Plants can achieve similar SLA or SRA/SRL values with a different investment in density or in leaf thickness/root diameter, which depends on species idiosyncrasies, their mycorrhizal association type and/or their environment (Poorter et al., 2009; de la Riva et al., 2016; John et al., 2017; Bergmann et al., 2020). As we have shown in this paper, analogous traits in roots and leaves generally display similar distributions in the two-dimensional PCA space. That is, the variation between root tissue density and diameter is similar to that between leaf density and thickness, irrespective of other traits considered, whether for roots or for leaves. This bivariate space has an underlying anatomical explanation; both SLA and SRA (or SRL) can be broken down into the product of leaf/root tissue density and leaf lamina thickness/root diameter (Ostonen et al., 2007; Poorter et al., 2009; see Appendix S3).

At the leaf level, SLA (or LMA) has been treated as a central attribute of plant strategies due to its strong correlation with photosynthetic and respiration rates, leaf nitrogen concentration and plant growth (Lambers and Poorter, 1992; Wright et al., 2004; Prieto et al., 2018). As a general trend, higher SLA across species is largely determined by a decrease in LD and thickness (Villar et al., 2013; de la Riva et al., 2016; John et al., 2017). But, at the same time, SLA is the inverse of the product of leaf density and thickness (Poorter et al., 2009) and thus, plants may achieve similar SLA values with different proportional investments in both traits, through different combinations in the size and number of cells and organelles (Pyankov et al., 1999; Villar et al., 2013). Overall, plants may increase their leaf thickness by having larger mesophyll cells, or by increasing the proportion of water contained within cells, both of which decrease leaf tissue density (Pyankov et al., 1999; Li et al., 2017). Within woody plants, larger mesophyll cell volumes increase leaf thickness, which may potentially reduce leaf density by diluting the influence of the denser tissues such as veins and the leaf cuticle (John et al., 2017). These authors also observed that the number of mesophyll cell layers influences only leaf thickness, while the cell dry mass density and air space fraction directly influenced leaf density. Therefore, the investment to build a given leaf area per unit dry mass (SLA or LMA) shows a strong dependency on both density and thickness (Gratani et al., 2018). In parallel, SRL (and also SRA) is the inverse of the product between root tissue density and root diameter (Ostonen et al., 2007). Thicker roots (i.e., with larger diameters) in *Pinus sylvestris* (L.) achieved lower root tissue densities by increasing the thickness of the cortex (Zadworny et al., 2017), while an increase of the aerenchyma in *Lotus glaber* (Mill.; Mendoza et al., 2005) and of schlerenchyma tissues in *Paspalum dilatatum* (Poir.; Vasellati et al., 2001) decreased their SRL. Wahl and Ryser (2000) observed that the proportion of cell wall in the stele was the main factor determining the interspecific variation in root density among herbaceous species. Furthermore, previous studies have shown contrasting plastic responses of root tissue density and root diameter to nutrient and water availability (Ryser and Lambers, 1995; Meier and Leuschner, 2008; Olmo et al., 2014), resulting in overall small variations in SRL despite large structural changes. Hence, different anatomical components may drive the trade-off between density and diameter/thickness, depending on the plant species and the environment.

Recent global analyses have proposed a positive correlation between root diameter and mycorrhizal colonization percentage (Bergmann et al., 2020), because thicker roots may enhance mycorrhizal associations by increasing the cortex thickness (Kong et al., 2019). These authors suggest that higher mycorrhizal dependency may help plants achieve a more efficient resource acquisition, suggesting deviations from a root economics spectrum of acquisitive to conservative belowground strategies. Thus, plants could optimize resource uptake by investing carbon either in abundant thin roots that efficiently explore the soil themselves or in fewer thicker roots that instead favor symbiosis with a mycorrhizal fungal partner. This interpretation assumes that larger root diameter enhances the plant’s acquisitive capacity *via* carbon investment in a mycorrhizal fungal partner in return for limiting resources like P or N (McCormack and Iversen, 2019). Mycorrhizal relationships can greatly increase the plant’s nutrient uptake; however, in addition to the need to build costly roots (e.g., large root diameters), they also add an extra cost associated with the transfer of C to the fungi to support the symbiosis (between 10 and 20% of net plant C assimilation on average; Allen et al., 2003). Therefore, a higher mycorrhizal root colonization percentage may enhance resource acquisition under poor soil fertility conditions without necessarily implying an acquisitive strategy (Navarro-Fernández et al., 2016). On the contrary, high soil fertility favors plant species with acquisitive root traits (de la Riva et al., 2018) but generally reduces mycorrhizal root colonization, likely because plants do not benefit from this association when soil nutrients are abundant and can be directly acquired by the root without the help of a mycorrhizal partner (Treseder, 2004). Hence, it is not clear how the mycorrhizal collaboration gradient proposed by Bergmann et al. (2020) relates to the differences in mass-normalized traits and root uptake efficiency between fast- and slow-growing species along the RES. A further analysis of our dataset, but using the same traits proposed in Bergmann et al. (2020) yielded a very similar pattern to that found by those authors; that is, a principal axis reflecting the trade-off between SRL and Rdi, and a second axis reflecting the trade-off between RN and root tissue density. However, when we replaced SRL by SRA in the trait space, we observed a reorientation of the bi-dimensional space with the main axis shifting towards the RES (axis 1; Figure 3B), and the abovementioned anatomical trade-off included in the second axis. In addition, when we analyzed leaf traits analogous to the root traits selected by Bergmann et al. (2020), a tight covariation between below- and aboveground traits became evident. Our findings suggest that some precaution is advisable with the interpretation of non-mass-based root trait variation (i.e., root diameter). Unfortunately, and similar to that stated for SRA earlier, there is not enough data available at the moment to carry out a comprehensive analysis to understand the covariation between analogous traits at root and leaf levels (e.g., only 59 species in the TRY database have information for all the traits included in Figure 3C). Up to date, attending to our knowledge about Mediterranean vegetation, our overarching perception is that the correlations of SLA and SRA/SRL with anatomy are only partially consistent with the intrinsic anatomical composition of each component separately, illustrating the axiom that “correlation does not imply direct causation” (e.g., John et al., 2017). We would like to stress the importance of an anatomically explicit approach, in which both density and thickness/diameter are taken into account, because plant growth is the increment in dry mass, volume, and length or area that results from the division, expansion, and differentiation of cells (Lambers and Oliveira, 2019). Unfortunately, the links between root anatomy and performance still lack a thorough understanding of the functional relationships between morphological features (Gonzalez-Paleo and Ravetta, 2018).

## Conclusion

In summary, fine roots display a wide diversity of forms and properties in Mediterranean vegetation, resulting in a multidimensional root trait space. Our findings highlight how the main trend of variation in this multidimensional root space is largely analogous to the main axis of the leaf economics spectrum. If we accept that plant performance is mainly modulated by mass-normalized leaf traits according to the LES theory, then we advocate that the same approach should be adopted for roots, for the sake of consistency. As we have demonstrated, mass-normalized root traits (especially SRA, RDMC, and RN) are good predictors of the RES in Mediterranean environments. However, wider analysis of morphological and physiological mass-normalized traits encompassing more vegetation types globally should be carried out in order to provide a general conceptual framework that confirms the proposed universal main trend of variation in roots related to the economics spectrum. In addition, we propose that a first step to analyze the trade-off between root density and diameter could be to follow an anatomical approach to understand how and why plants build their fine roots. Thus, further anatomical studies similar to those carried out for leaves (i.e., Villar et al., 2013; de la Riva et al., 2016; John et al., 2017; Li et al., 2017; Gratani et al., 2018), would be necessary to obtain solid conclusions on the role of root anatomical tissues in defining the SRA or SRL of fine roots, which could help clarify the discrepancies between tissue density and root diameter.

## Data Availability Statement

The raw data supporting the conclusions of this article will be made available by the authors, without undue reservation.

## Author Contributions

ER, JQ, and IP conceived the ideas, designed the study, and wrote the first draft. ER, IP, JGD, SM, TM, and IP-R conducted fieldwork. ER performed statistical analyses. All authors contributed to the article and approved the submitted version.

## Funding

This work was financially supported by the German Research Foundation (ECOFUMER, GZ: GA 2899/3-1), Spanish Ministry of Science and Innovation (grant no. CGL2017-82254-R-INTARSU), ECO-MEDIT (CGL2014-53236-R), the projects “Ecología Funcional de los Bosques Andaluces y Predicciones Sobre sus Cambios Futuros (For-Change; UCO-FEDER 18 REF 27943 MOD B; Spain) and Funcionalidad y Servicios Ecosistémicos de los Bosques Andaluces y Normarroquíes: Relaciones con la Diversidad Vegetal y Edáfica Ante el Cambio Climático” (P18-RT-3455) by Junta de Andalucía (Spain), and the Seneca Foundation (project 20654/JLI/18), all cofinanced by European FEDER funds.

## Conflict of Interest

The authors declare that the research was conducted in the absence of any commercial or financial relationships that could be construed as a potential conflict of interest.

## Publisher’s Note

All claims expressed in this article are solely those of the authors and do not necessarily represent those of their affiliated organizations, or those of the publisher, the editors and the reviewers. Any product that may be evaluated in this article, or claim that may be made by its manufacturer, is not guaranteed or endorsed by the publisher.
