# Supplementary material for: The Economics Spectrum Drives Root Trait Strategies in Mediterranean Vegetation
Source: Front Plant Sci. 2021 Nov 23;12:773118. doi: 10.3389/fpls.2021.773118 (PMC8649719; doi:10.3389/fpls.2021.773118)
Supplement: Supplementary file 1 [file Data_Sheet_1.PDF]

Supplementary Information

Appendix S1. List and content of datasets used in this study. Abbreviations are as in Figure 1 in the main body of the manuscript.

Reference	Obs.	Sp.	Life Forms	Habitat	Localization	Annual Precipitation (mm)	Mean Annual Temperature (ºC)	Traits	Data availability
de la Riva et al. 2016a,b,c	75	38	W	Dry and Riparian Forest	Sierra Morena	655	17.5	SRA,SRL, RTD, Rdi, RDMC, SLA, LDMC, Lthick, LD[1]	Dryad Digital Repository http://dx.doi.org/10.5061/dryad.dr275
de la Riva et al. 2018a,b	80	80	W	Arid shrubland<br>Semiarid-shrubland<br>Sub-humid Forest	Cabo de Gata Natural Park<br>Doñana National Park<br>Alcornales Natural Park	240<br>560<br>1056	17.8<br>16.5<br>16.5	SRA,SRL, RTD, Rdi, RDMC, SLA, LDMC, Lthick, LD[1]	Root traits in Table S2. Supporing information https://doi.org/10.1007/s11104-017-3433-4 Leaf traits in Dryad
de la Riva et al. 2021	73	60	W	Regions from de la Riva 2016 and 2018		-	-	Org. N, Min.Conc, RC	Dryad Digital Repository
de la Riva et al. 2019	110	110	W/H	Coastal Dunnes	Coast of Huelva	560	16.5	SRA,SRL, RTD, Rdi, RDMC, SLA, LDMC, Lthick[2], LD[1]	Unpublish
Marañon et al. 2021	7	7	W	Dry and Riparian Forest	Guadiamar Green Corridor	450	17	SRA,SRL, RTD, Rdi, RDMC, RN, RC, SLA, LDMC,	Unpublished
Galan Díaz 2021 (PhD Thesis)	178	107	H	Grasslands	Sierra Norte de Sevilla<br>Alcornales Natural Park	617<br>796	17<br>16	SRA,SRL, RTD, Rdi, RDMC, SLA, LDMC, Lthick[2], LD[1]	Unpublished
Unpublish data from de la Riva	27	27	H	Temporal Lagoon	University of Cordoba	518	17.9	SRA,SRL, RTD, Rdi, RDMC, SLA, LDMC, Lthick[2], LD[1]	Unpublished
Unpublish data from de la Riva	14	14	W	Arid Shrublands	Monegros Natura Park	390	14.8	SRA,SRL, RTD, Rdi, RDMC	Unpublished
Unpublish data from de Tomás Marín	13	13	W	Sub-humid Forest	Hayedo de Montejo	954	9.5	SRA,SRL, RTD, Rdi, RDMC, SLA, LDMC, Lthick, LD[1]	Unpublished
Unpublish data from Prieto	50	33	W	Arid Shrublands<br>Semiarid Shrublands<br>Dry Forest<br>Subhumid Forest	Pulpí village<br>Sierra Espuña<br>Sierra de Alcaraz<br>Sierra del Segura	203<br>334<br>382<br>746	19.9<br>17.7<br>16.4<br>14.4	SRA,SRL, RTD, Rdi, RDMC, SLA, LDMC, Lthick[2], LD[1]	Unpublished

[1] Lthick was calculated as 1/(SLA+LDMC) (Vile et al. 2005).
[2] LTD was calculated as the ratio of LMA and LVA (leaf thickness) (Witkowski et al. 1991).

Vile, D., Garnier, E., Shipley, B., Laurent, G., Navas, M. L., Roumet, C., ... & Wright, I. J. (2005). Specific leaf area and dry matter content estimate thickness in laminar leaves. *Annals of botany*, 96(6), 1129-1136.
Witkowski, E. T. F., & Lamont, B. B. (1991). Leaf specific mass confounds leaf density and thickness. *Oecologia*, 88(4), 486-493.

Appendix S2. Principal Component Analysis (PCA) on trait means in 318 Mediterranean plant species with phylogenetically independent contrasts (seedless plants including -*Equisetum ramosissimum* and *Pteridium aquilinum*- were excluded from the analysis because they are not available in ALLMB tree). The phylogenetic tree of the studied species was obtained from the ALLMB tree (Smith & Brown 2018), available in https://github.com/FePhyFoFum/big_seed_plant_trees. The phylogenetic PCA was carried out with the "phyl.pca" function implemented in the library RPANDA (Morlon et al. 2016).

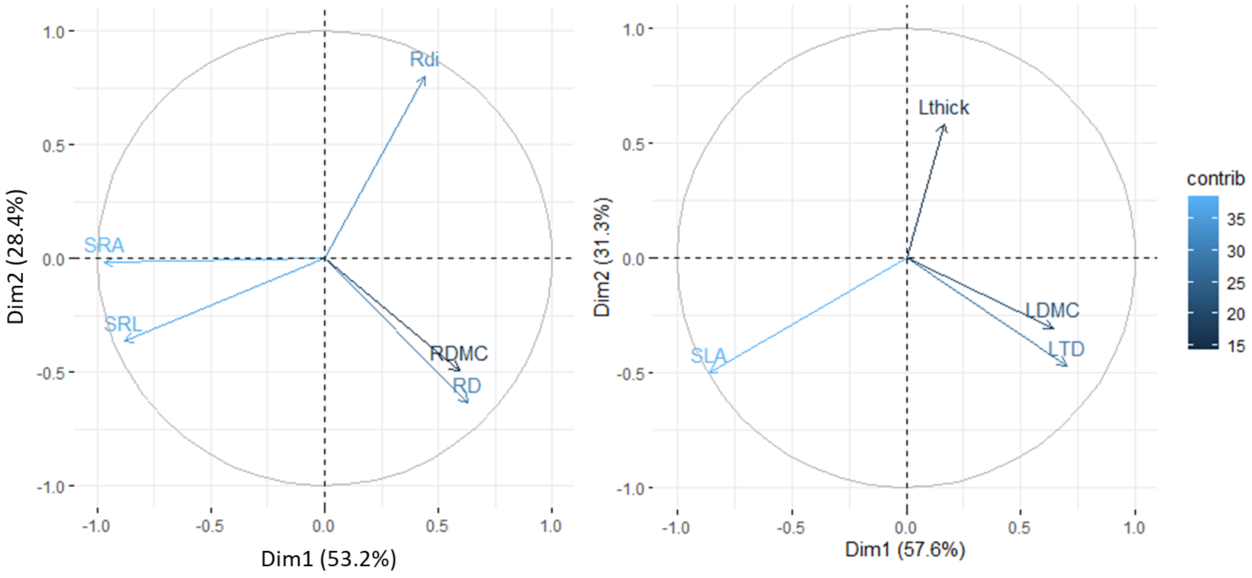

Smith, S. A., and J. W. Brown. 2018. Constructing a broadly inclusive seed plant phylogeny. *American Journal of Botany,* 105(3): 1–13.

Morlon, H., Lewitus, E., Condamine, F., Manceau, M., Clavel, J. & Drury, J. (2016). "RPANDA: an R package for macroevolutionary analyses on phylogenetic trees." Methods in Ecology and Evolution, 7, 589-597. R package version 1.4, https://CRAN.R-project.org/package=RPANDA.

Appendix S3. Breakdown of SLA, SRL and SRA into its components

SLA

The specific leaf area (SLA) is the ratio of leaf area (A) and leaf dry mass (M).

$$SLA = \frac{A}{M}$$ (Equation 1)

Leaf mass density (LTD) is the ratio of leaf dry mass and leaf volume (V)

$$LTD = \frac{M}{V}$$ (Equation 2)

Then $M = LTD * V$ (Equation 3)

Using equation 1 and equation 3 we have:

$$SLA = \frac{A}{LTD*V}$$ (Equation 4)

Leaf volume is the product of leaf area and leaf thickness (Lthick)

$$V = A * \ Lthick$$ (Equation 5)

Using equation 4 and equation 5 we have:

$$SLA = \frac{A}{LTD*A*Lthick}$$ (Equation 6)

Then

$$\boldsymbol{SLA = \frac{1}{LTD*Lthick}}$$ (Equation 7)

SRL

Specific root length (SRL) is the ratio of root length (L) and root dry mass (M). We follow Ostonen et al. (2007) and Olmo et al. (2014)

$$SRL = \frac{L}{M}$$ *(Equation 8)*

Root tissue mass density (TMDr) expresses the ratio of root dry mass to root volume (V):

$$TMDr = \frac{M}{V}$$ *(Equation 9)*

From equation 9 we can solve *M*: $M = TMDr \times V$ *(Equation 10)*

Root volume (V) also can be expressed as: $V = \pi \times (\frac{RD}{2})^2 \times L$ *(Equation 11)*

where RD is the root diameter

Simplifying equation 11: $V = (\frac{\pi}{4}) \times RD^2 \times L$ *(Equation 12)*

Replacing *V* in equation 10 by equation 12:

$$M = TMDr \times (\frac{\pi}{4}) \times RD^2 \times L$$ *(Equation 13)*

Replacing *M* in equation 8 by equation 13

$$SRL = \frac{L}{TMDr \times (\frac{\pi}{4}) \times RD^2 \times L}$$ *(Equation 14)*

Simplifying equation 14:

$$SRL = \frac{1}{TMDr \times RD^2} \times (\frac{4}{\pi})$$ *(Equation 15)*

SRA

Specific root area (SRA) is the ratio of root surface area (SA) and root dry mass (M).

$$SRA = \frac{SA}{M}$$ *(Equation 16)*

Using equation 10

$$SRA = \frac{SA}{TMDr * V}$$ *(Equation 17)*

Root volume is the product of root area and root length (L)

$V = A * L$ *and* $V = \pi * \left(\frac{D}{2}\right)^2 * L$ *(Equation 18)*

Surface area is $SA = D * \pi * L$ *(Equation 19)*

Using equation 18 and 19 equation on equation 17 we have:

$$SRA = \frac{D * \pi * L}{TMDr * \pi * \left(\frac{D}{2}\right)^2 * L}$$ *(Equation 20)*

Simplifying equation 20:

$$\boldsymbol{SRA = 4 * \frac{1}{TMDr * D}}$$ *(Equation 21)*
